# An in vitro model to explore subchondral perfusion and intraosseous pressure

**DOI:** 10.1186/s40634-019-0207-3

**Published:** 2019-08-30

**Authors:** Michael Beverly, David Murray

**Affiliations:** Nuffield Department of Orthopaedics, Rheumatology & Musculoskeletal Sciences, University of Oxford, Oxford, UK

**Keywords:** Intraosseous pressure, Perfusion, Physiology, Subchondral, In vitro model, Tourniquet

## Abstract

**Background:**

Little is known about subchondral perfusion physiology. We developed a 3Rs (Replace, Reduce, Refine) compliant in vitro calf foot model to explore perfusion and intraosseous pressure (IOP).

**Methods:**

Calf feet were catheterised and perfused with serum. IOP was measured at three sites, the metacarpal diaphysis (MCD), metacarpal subchondral epiphysis (MCS) and proximal phalanx diaphysis (PPD) using intraosseous needles with pressure transducers and digital recorders. Fresh (< 4 h post mortem) and old feet (> 4 h post mortem) were perfused at different pressures, with and without a proximal tourniquet.

**Results:**

There was a wide range in basal IOP with a mean IOP of 30.0 mmHg, SD 14.4, range 7.6 mmHg to 52.7 mmHg (*n* = 40 records) in 15 subjects. There was no significant difference between the three sites tested (*p* = 0.54, 0.12 and 0.051). At each individual site IOP correlated with perfusion pressure (r = 0.993). With a proximal venous tourniquet, IOP increased from 15.1 mmHg (SD 11.3 mmHg) to 44.9 mmHg (SD 24 mmHg), *p* < 0.0001, *n* = 9. Filling and emptying curves during perfusion and with using a tourniquet were similar, indicating that the model behaves in an elastic hydrodynamic manner. In fresh feet IOP peaked after about 1 min irrespective of perfusion pressure, possibly due to auto regulation. Older feet showed a continuously rising IOP and became oedematous. There was no significant difference in IOP between fresh and old feet perfused with serum at 150 cms pressure for 1 min.

**Conclusion:**

Though basal intraosseous pressure varies, IOP behaves predictably. IOP measurements reflect the perfusion microclimate at the individual needle tip. This 3Rs compliant model will be used for further exploration of subchondral perfusion physiology with loading.

## Background

Intraosseous pressure (IOP) and bone blood flow have been studied by authors interested in bone circulation, bone diseases and bone pain for more than 70 years (Arnoldi et al. 1980; Lemperg and Arnoldi 1978; Liu and Ho 1991; Owen et al. 1980; Termansen and Okholm 1976; Wilkes and Visscher 1975). Measurement techniques have varied. There has been difficulty in establishing reliable values for IOP (Arnoldi et al. 1980; Bourne 1972; Bouteiller et al. 1983; Bryant 1983; De Lorenzo et al. 2014). It is generally assumed that IOP is due to a background venous pressure or intrinsic tissue pressure and that the same pressure is present throughout the bone (Ficat 1985; Frascone et al. 2015). There has been limited progress in understanding IOP perfusion since Azuma reported IOP fluctuation in a rabbit model in 1964 (Azuma 1964; Hungerford and Lennox 1985). IOP has generally been found to be raised in bone diseases such as osteonecrosis and after steroid use. Avascular necrosis of the hip remains a significant problem (Lamb et al. 2019). A raised IOP has been associated with pain in osteoarthritic joints, chondromalacia patellae and with cartilage degeneration (Kofoed 1986). IOP may also be important in driving fluid through canaliculi, hence governing osteocyte activity and bone turnover (Cardoso et al. 2013; Verbruggen et al. 2014). Steroid induced models of avascular necrosis have been developed in order to study IOP and its treatments (Drescher et al. 2001; Miyanishi et al. 2005). Clinical measurement of IOP in man has offered variable results (Salzman et al. 2017). Ficat developed a technique for the ‘functional exploration’ of bone in patients with early osteonecrosis (Ficat 1985). However, the factors that control IOP at rest and the physiology of subchondral bone circulation remain largely unknown. Previous authors have found pulsation and even a respiratory wave in IOP recordings (Beverly et al. 2016; Beverly and Murray 2018). More recently there has been interest in IOP during exsanguination in a porcine model used to study intraosseous perfusion in emergency resuscitation situations (Frascone et al. 2015). Simkin has suggested that subchondral marrow fat distributes subchondral pressure rather than the trabeculae taking the whole load (Simkin 2018). The rationale for this study was to develop an in vitro model which was compliant with the Animals (Scientific Procedures) Act 1986 (ASPA) 3Rs (Replace, Reduce and Refine) for animal research. The model would be used to study the IOP and perfusion physiology of cancellous bone by altering perfusion pressures and by use of a tourniquet.

## Methods

### Model preparation

Freshly culled male Friesian calves 1–2 weeks of age and weighing 50–80 kg were used. The fore feet were sectioned above the carpus. The radial artery was immediately catheterised with a 16 gauge 12″ Teflon catheter (E-Z Cath, Desert Pharmaceutical, Sandy, Utah) and the foot was rinsed with 120 ml of warmed Krebs-Henseleit (K-H) solution (Sigma Pharmaceuticals PLC, Watford, UK). The catheters were inserted by hand to the point at which they could no longer be advanced, that is to about 6″. They were then withdrawn for a few millimetres to allow perfusion without obstruction. The feet were set up in a water bath at 37 °C within one to 2 h. These were referred to as ‘fresh feet’. Other feet were kept at room temperature until later the same day or overnight at 5 °C or frozen at − 80 °C with rewarming in a water bath for 2 h before being used. These were called ‘old feet’. The water bath had a circulating pump (Fluval FLU.U1 model C-48020 from Rolf C. Hagen UK Ltd, Castleford, UK) and a heater to maintain the temperature at 37 °C (Aqua One 100-W IPX8, Aqua Pacific UK Ltd., Southampton,). A room thermometer confirmed that the temperature remained stable within a degree for the duration of the experiment. A doubled string tourniquet was applied proximally across the carpal joint and was tensioned by twisting in forceps. It could be applied and released without moving the foot in the water bath. Serum was prepared by collecting fresh calf blood, centrifuging at 10,000 rpm for 10 min at 4 °C and taking the supernatant fluid though a filter into plastic storage bottles kept in a − 80 °C fridge. The serum was warmed and passed through an IV line which lay in the water bath before entering the foot. The calf serum was perfused from a fixed height above the foot using a 1 litre Mariotte flask at 50, 100 or 150 cms. These heights equate to pressures of 37, 74 and 110 mmHg. K-H saline filled intraosseous needles (Rosenthal Bone Marrow Biopsy Needle, 16 adult, Luer lock fitting, 16 swg 1.6 mm × 35 mm Dixons Surgical Instruments Ltd., Wickford, UK) were inserted by hand percutaneously into the metacarpal diaphysis (MCD), metacarpal subchondral (MCS) bone or mid proximal phalanx diaphysis (PPD). The needles were placed in the centre of the cancellous bone or within a few millimetres of the subchondral surface. The trochar was withdrawn and the needles were filled with K-H saline and connected to catheter tip pressure transducers (Model TT Luer, Gaeltec Devices Ltd., Isle of Skye, UK). Care was taken to avoid forced ‘clearance’ of the needles. The pressure transducers were connected to a Gaeltec S7d transducer amplifier and an ADC-20 Picolog High Resolution Data Logger (Pico Technology, Saint Neots, UK). Data was recorded continuously using a Picolog Recorder program on a Prodesk Hewlett-Packard PC. The pressure transducers were calibrated against a 100 mmHg sphygmomanometer at the start of each experiment and the system was zeroed before each fresh run. The formula for converting the millivolt recorded values to mmHg was applied to all readings universally and assumed that 150 cm H_2_O is equivalent to 110 mmHg.

One of the four channels was used for recording the perfusion pressure at the point of entry to the arterial catheter. Recordings were made from up to three separate site intraosseous needle channels at one per second for up to an hour. For this work 8 feet were tested fresh within 2 h of culling. Seven feet were tested after more than 4 h, known as old feet. In addition to the perfusion studies the effect of a tourniquet was explored in 9 feet; 6 fresh and 3 old.

### Experimental plan

After initial trials to develop the model, experiments were conducted to establish the IOP response to static perfusion at different sites, times and pressures in fresh and old feet, with and without a tourniquet.

### Statistics

Results were expressed as means, standard deviations and ranges. Student’s t-test was used to determine if there were significant differences. When each subject was used as its own control, paired tests were used. Otherwise unpaired t-tests were used. The Pearson test was used to assess correlations with *p* < 0.05 considered to be statistically significant.

## Results

### Perfusion pressure range

Using the same methods and under a constant perfusion pressure of 150 cm water for 60 s there was a wide range of basal IOPs. There were 15 subjects, 8 fresh and 7 old feet, with 40 separate site IOP recordings. There was a mean IOP of 30.0 mmHg with a wide variation (SD 14.4 mmHg, range 7.6 mmHg to 52.7 mmHg). There was no significant difference between the fresh (32.4 mmHg, SD 17.6 mmHg) and old (27.3 mmHg, SD 10.4 mmHg) feet at this point, t-test *p* = 0.513.

### Site differences

**Fig. 1:**
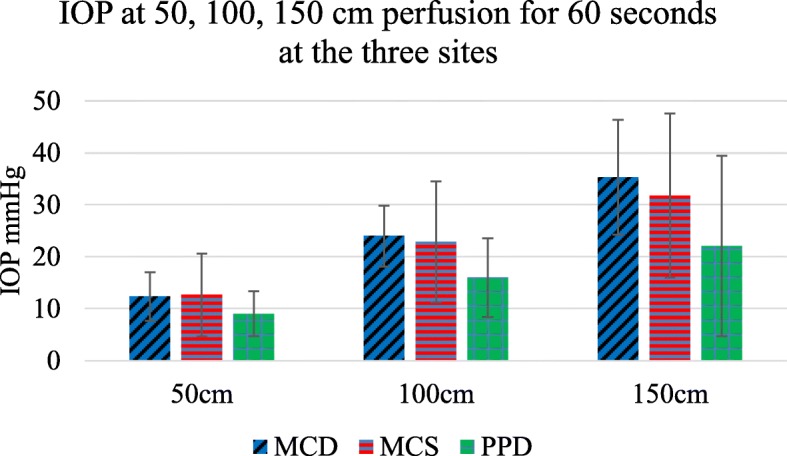
Difference in IOP at 60 s perfusion at the different sites tested, Blue - MCD - metacarpal diaphysis, Red - MCS - metacarpal subchondral, Green - PPD - proximal phalanx diaphysis *n* = 40 sites in 15 subjects. For each individual site IOP was correlated with perfusion pressure, *r* = 0.993, error bars SD

There was no significant difference in IOP between the sites tested. Forty different IOP recordings were obtained from the three different sites in 15 calf feet under the same perfusion conditions (50, 100 and 150 cm serum for 60 s). There were 10 separate metacarpal diaphysis recordings (MCD), 18 metacarpal subchondral recordings (MCS) and 12 from the diaphysis of the proximal phalanx (PPD) (Fig. 1). There were no significant differences between the sites at 150 cm pressure perfusion for 60 s, MCD to MCS *p* = 0.54, MCS to PPD *p* = 0.124 and MCD to PPD *p* = 0.051.

At each individual site IOP was strongly correlated with perfusion pressure. Perfusion with serum at 50 cms, 100 cms and 150 cms pressure for 60 s resulted in proportionately higher IOP, *r* = 0.9993.

### IOP rise in perfusion pressure with time

**Fig. 2:**
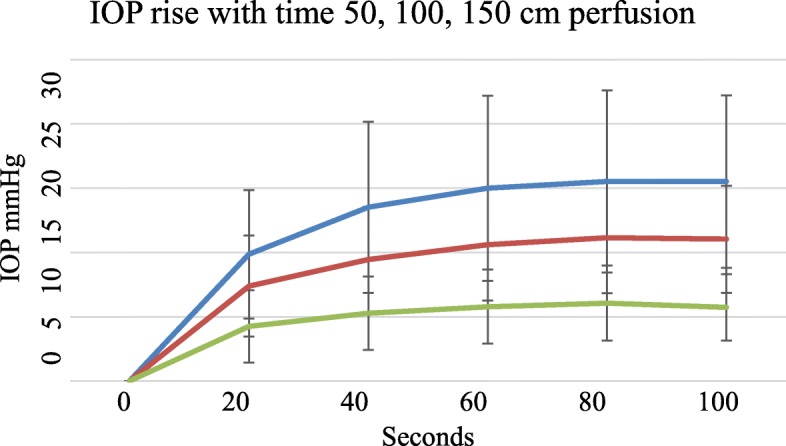
Rise in IOP with time among 15 subjects at different perfusion pressures, Blue - perfusion at 150 cms, Red – perfusion at 100 cms and Green – perfusion at 50 cms pressure, error bars SD

Initial IOP was always zero both in freshly prepared and older feet. There was no intrinsic or background IOP. IOP rose with perfusion as shown. With perfusion at higher pressures there were proportionately higher pressure filling curves (Fig. 2).

### Tourniquet response

**Fig. 3:**
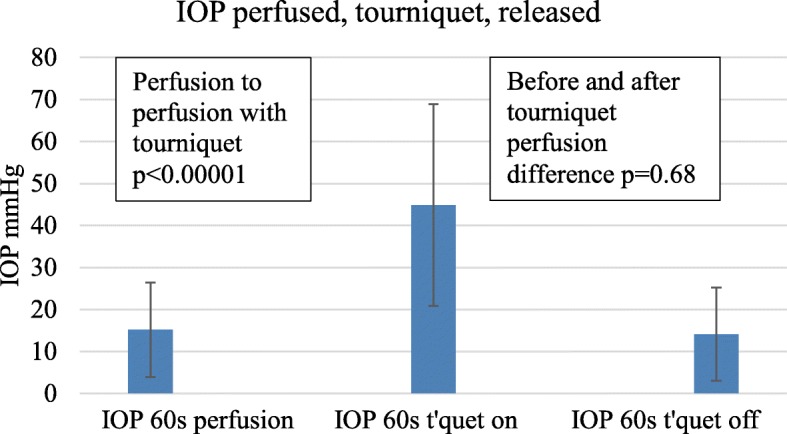
Additional rise in IOP with a proximal tourniquet for 60 s and effect of removal of the tourniquet after a further 60 s with continued perfusion of serum at 150 cms pressure, error bars SD

A tourniquet was used in a group of nine subjects (6 fresh and 3 old feet). After perfusion at 150 cm serum for 60 s the IOP was a mean of 15.14 mmHg (SD 11.3 mmHg). When a venous tourniquet was applied proximally while perfusion at 150 cms continued, the IOP rose to 44.9 mmHg (*P* < 0.0001) after a further 60 s. On removal of the tourniquet but with continuing perfusion at 150 cm serum the IOP fell back to a mean of 14.1 mmHg (SD 11.1) after 60 s (*p* < 0.0001) (Fig. 3). The difference in IOP before and after use of a tourniquet was not significant (*p* = 0.68).

### Pressure rise and fall curves

**Fig. 4:**
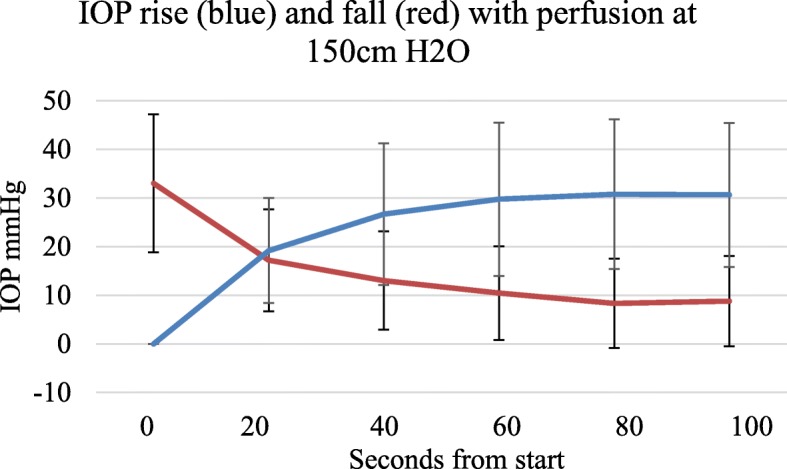
Relative rates of rise and fall with perfusion at 150 cm serum, *n* = 15, error bars SD

There was a rise in IOP with perfusion for about a minute and a fall with cessation of perfusion as shown in Fig. 4. Where there had been no previous perfusion during experimental set up the first part of the filling curve to about 10 mmHg represents an initial filling or charging of the system. Subsequent emptying and refilling cycles at minute intervals had symmetrical curves. Furthermore, the filling and emptying pressures on application and removal of a tourniquet at minute intervals were symmetrical.

### Fresh / old filling difference

**Fig. 5:**
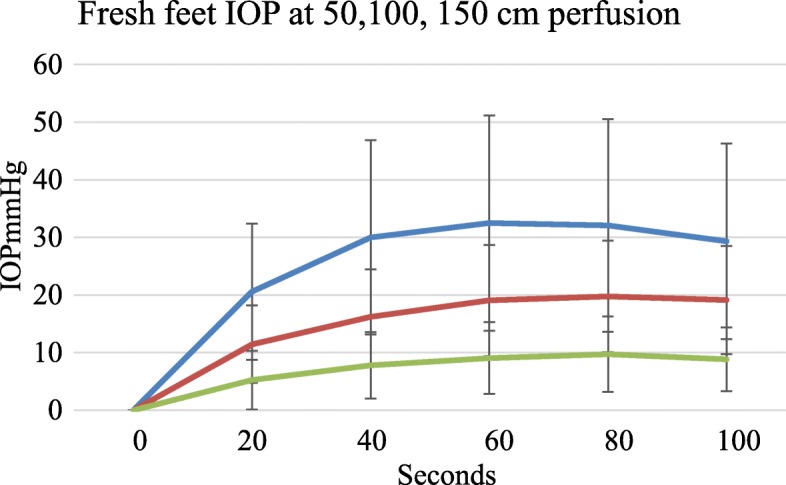
For fresh feet IOP peaked at about 1 min with no further increase up to 100 seconds (t-test *p* = 0.4). Blue = 150 cm, Red = 100 cm, Green = 50 cm perfusion pressure, error bars SD.

**Fig. 6:**
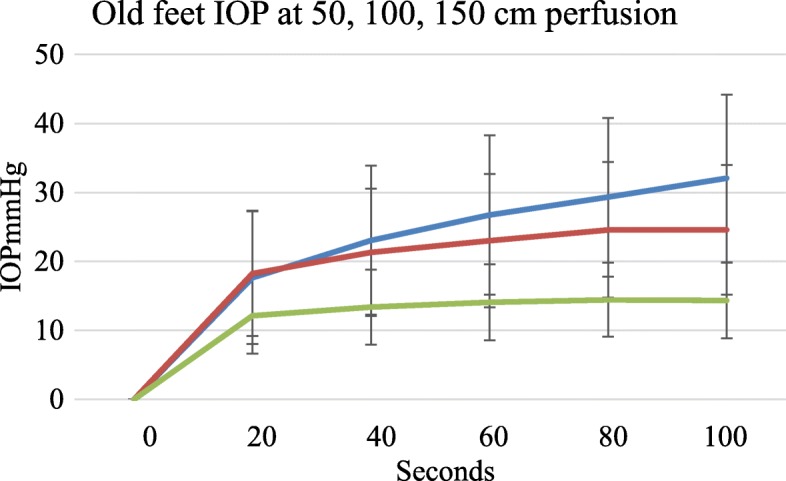
Older feet had a different filling curve profile with continued perfusion. They become increasingly oedematous with no obvious IOP end point. Blue = 150 cm, Red = 100 cm, Green = 50 cm perfusion pressure, error bars SD

There were differences in IOP filling curves between fresh feet less than 4 h old and feet more than 4 h old. With low pressure perfusion fresh feet had lower IOP for the same perfusion pressure than feet more than 4 h old. The difference was less marked at higher perfusion pressures as shown in Figs. 5 and 6 and Table 1.

**Table 1:** Difference between fresh and old feet. At 150 cms perfusion pressure the fresh feet showed no difference in IOP between 60 to 100 s (32.4 mmHg to 29.8 mmHg, *p* = 0.4)

T-test Fr/Old	20 s	40 s	60 s	80 s	100 s
150 cm	*P* = 0.40	*P* = 0.13	*P* = 0.26	*P* = 0.58	*P* = 0.57
100 cm	*P* < 0.02	*P* = 0.10	*P* = 0.24	*P* = 0.16	*P* = 0.10
50 cm	*P* < 0.0001	*P* < 0.01	*P* < 0.02	*P* < 0.03	*P* < 0.0001

There was no statistical difference between Fresh and Old feet perfused at 150 cms serum for 60 s (*p* = 0.256) and 100 s (*p* = 0.568)

Fresh feet perfused at a steady pressure appeared to stabilise their IOP after about 1 min as in Fig. 5.

Older feet at lower perfusion pressures had higher IOPs than fresh feet. Continued perfusion caused an indefinite slow rise in IOP shown in Fig. 6. The older feet gradually became visibly oedematous. From 60 to 100 s older feet perfused at 150 cm pressure showed a continuing rise in IOP (27.3 mmHg to 32.4 mmHg, *p* < 0.002), Fig. 6.

## Discussion

Our work demonstrates a wide variation for individual IOP measurements obtained under similar circumstances. No other physiological parameters would be expected to show such variation in a group of otherwise nearly identical experiments. While previous authors have struggled to identify a normal IOP, it is generally held that bone has an IOP which is a constant, present throughout the bone and in healthy bone is approximately a third of systolic pressure or about 30 mmHg (Owen et al. 1980; Wilkes and Visscher 1975; Bourne 1972; Salzman et al. 2017). We consider that our findings point to there being an alternative explanation for this variation. We believe that the variation is due not to some significant physiological difference between subjects or within bones but to the random nature of the vessels encountered at the needle tip even with a standardised needle insertion technique. We make the assumption that the IOP recorded will always be that of the highest pressure vessel present at the needle tip. Where a small artery is struck a relatively high IOP is recorded. Where capillaries or veins, fat and trabeculae are penetrated then a lower IOP is found. It is surprising that for several decades it has been assumed that cancellous bone has an intrinsic or constant background IOP and that the IOP may be measured by inserting a needle into a solid organ (Wilkes and Visscher 1975; Ficat 1985; Hungerford and Lennox 1985; Ficat 1985). All other pressure measurements in the body rely on either catheterisation of an artery or vein or measurement of pressure within defined fluid spaces such as in the brain ventricle or bladder. It is only in bone that it has been assumed that an intraosseous needle will give a reliable pressure reading and one representative for the whole organ. We consider that the wide distribution of values found in spite of a similar technique supports the idea the IOP is a measure of pressure only at the needle tip blood pool which is itself dependent on the local vessels contacted (Beverly et al. 2016).

The proximal end of the specimen was open at the amputation site. Even with the open proximal end of the foot a reasonable IOP is generated within a minute or so indicating that there is some form of filling of the foot and bone contained within. There must be some resistance or obstruction to free exit of the perfusing serum. It remains uncertain what proportion of the IOP is due to arterial supply pressure and what proportion represents venous back pressure. It was not technically possible to catheterise the several superficial draining vessels from the limb.

Site differences appeared to be minimal. In the calf there is an epiphyseal plate between the metacarpal diaphysis and the epiphysis. There is little or no flow across the epiphyseal plate. The two areas are likely to be functionally isolated from each other in a vascular sense. With respect to the proximal phalanx, although there is a single proximal epiphysis, our needles were placed in the mid shaft or diaphysis cancellous bone region. In effect in our model we were looking at three separate bones each with their own circulation. For the purposes of this work the different sites produced no significant difference in IOP. We consider that the similar IOPs at different sites allows us to pool results from those areas for further analysis. While variation in IOP between subjects was considerable, at any single needle site in any subject there was a close correlation between IOP and the perfusion pressure. Other authors have found that IOP in healthy volunteers varies (Salzman et al. 2017; Frascone et al. 2014) We consider that even though there is a wide range of starting pressures our model is reliable in terms of testing perfusion responses at an individual needle tip level.

In terms of perfusion filling time, we were not surprised to find that in our model the starting or initial IOP was zero. In vivo when a needle penetrates a bone an IOP is instantly recordable but in vitro, none was found. There was no evidence of a background intrinsic pressure or tissue turgor even in fresh specimens (Hungerford and Lennox 1985; Ficat 1985; Hughes and Mccarthy 1993). In order to register an IOP the foot required initial ‘filling’ or charging with serum. Given the dimensions of the system our results show that perfusion for about a minute was sufficient to load or charge the foot and the contained bone, whatever the perfusion pressure as in Fig. 4. While pressures of 50 cms and 100 cms water equivalent to 37 mmHg and 74 mmHg were tried we believe that the 150 cms height pressure equivalent to 110 mmHg is the most physiological pressure to use in this model. As our graphs show the perfusion filling time was about a minute and we took this for a reasonable duration of observation or filling before recording results both with perfusion and tourniquet measurements.

In this model the proximal tourniquet did not prevent arterial perfusion. The catheter was a polythene tube lying within the artery. It was not crushed by the tourniquet. This allowed perfusion to continue. It is not clear to what extent the IOP is due to arterial supply or venous back pressure. Probably both contribute to IOP in different proportions. If the initial IOP is based mainly on arterial supply it might be expected that after application of a proximal venous tourniquet the limb would fill to 150 cm water pressure IOP in all areas but this is not the case. An explanation might be that in any vascular ‘tree’ the pressure will fall further down the capillary tree. There is a stepping down in pressure though the smaller vessels. Even if venous outflow is completely prevented there is no compelling reason for the IOP back pressure to ever fully achieve the arterial filling pressure. Much the same logic applies to venous drainage or back pressure with loss of the perfusion pressure supply. The IOP never quite approached zero indicating that there is a residual tissue or venous fluid resistance to out flow even in these amputated specimens.

During constant perfusion at a steady 150 cms pressure the use of a tourniquet for 60 s caused a further rise in IOP which, on removal of the tourniquet, was fully reversed within a further 60 s. In terms of flexibility or elasticity the model demonstrated equal ‘filling’ and ‘emptying’ curves and we therefore assume that the system is hydrodynamically ‘elastic’. During the initial experimental set up, perhaps up to an hour or two post-mortem, any fluid in the vascular system had fully drained away. The first filling or charging of the limb results in a slightly different curve to subsequent filling curves where the limb has already been filled. Thereafter the filling and emptying curves were symmetrical indicating that the system was elastic.

We have shown that at any individual needle tip site there is a close relationship between IOP and the perfusion pressure in spite of the wide range of initial values encountered. This is perhaps unsurprising and confirms that we are dealing with a sampling of individual points along a vascular tree with IOP reflecting the pressure in the serum pool at the needle tip rather than measuring a constant pressure for the whole tissue or organ.

In terms of pressure regulation, we found that there was a difference between feet in filling curves depending on their age or presumed viability. Feet up to 4 h post-mortem filled as expected but after 60–80 s demonstrated a static IOP as in Fig. 5. This contrasts with the pressure curves seen in older and probably non-viable tissue. An auto regulatory response is recognised to occur in the absence of neural and hormonal influences and is a property intrinsic to the organ. While well documented in the brain or kidney it has not been reported in bone. We consider that the similarity in response in the fresh and old feet at 150 cms perfusion for 60 s offers a basis for the use of both fresh and older feet in future loading studies.

Our model is not an isolated bone but an amputated limb. We measured IOP only in the bone but this was contained in the surrounding bag of flexible soft tissue. We see no reason for bone to require an intrinsic autoregulation mechanism and in any event are unable to exclude that regulation, if present, may be occurring in the surrounding soft tissues. Older feet did not show regulation, as in Fig. 6. In the older feet IOP continued to rise slowly even after 500 s but never reached arterial supply pressure. After prolonged perfusion the feet became visibly oedematous. However, in our model there was no statistical difference between fresh and old feet after 1 min of perfusion with serum at 150 cms pressure.

The model is potentially more complex. We understand bone to be a relatively rigid structure or enclosed box which has a low blood content compared to surrounding tissues. There might be almost instant filling of the bony box if it were to be directly connected to the arterial supply or oblique nutrient vessels. In fact, in our model the bone lies in a surrounding bag of softer fore foot tissue. This probably takes the majority of the blood flow and requires an appreciable time to fill and empty both with and without a tourniquet.

There are several possible limitations in this work. The calves were all male Friesian breed animals of similar but not identical age and weight. They were probably more similar than any group of patients might be. They were otherwise healthy but the bone was of a juvenile pattern with cartilaginous epiphyses present. We used the forefeet, often in pairs but specific pairing was not recorded. The calf has a fused third and fourth metacarpal which becomes bifid at the distal metacarpal and the phalanges and hoof. There is no direct human equivalent but the specimens resembled the human forearm and wrist in size and weight. The feet were catheterised through the equivalent of the radial artery and rinsed out. The exact depth of insertion of the catheter varied but was to about 6″ or well into the proximal third of the metacarpal. Flushing was carried out immediately using a 60 ml Luer lock syringe and two fills of warmed Krebs Henseleit solution by hand. The irrigation pressure may have varied accordingly. The emerging perfusate was almost but not completely clear of blood. Needle insertion was by hand and the sites could not be identical. Placement within a few millimetres of the subchondral surface or the central diaphysis was achievable. In spite of practice with insertion, connections and setting up of the pressure transducers the experiment took time and could not be guaranteed to work on every occasion. A doubled string tourniquet was applied at the level of the carpus. The tourniquet did not crush the supplying artery which contained the polythene catheter. The perfused serum varied in colour between batches from straw colour to a medium haemoglobin pink. Of all attempted recordings some 70–80% were successful. Needle leakage, obstruction, bubbles and unknown transducer, recorder and data collection failures appeared to account for the others. In our analysis all useable records for each experiment were included. No outliers were excluded.

## Conclusions

In conclusion the object of this work was to develop an in vitro 3Rs compliant model to explore IOP and bone perfusion physiology. We have achieved that. IOP measurements vary due to differing conditions at the needle tip rather than there being a significant physiological difference. Bone behaves as a perfused tissue and demonstrates the expected IOP responses to hydrodynamic changes and the use of a tourniquet. Our model offers a basis for further in vitro work on bone perfusion physiology and IOP during loading.
